# Robert Burns (Poetry)

**Published:** 1881-02

**Authors:** Henry W. Longfellow

**Affiliations:** Harper’s Magazine


### ROBERT BURNS.

I see amid the fields of Ayr
A plowman, who, in foul or fair,
Sings at his task,
So clear we know not if it is
The laverock's song we hear or his,
Nor care to ask.

For him the plowing of those fields
A more ethereal harvest yields
Than sheaves of grain;
Songs flush with purple bloom the rye,
The plover's call, the curlew's cry,
Sing in his brain.

Touched by his hand, the wayside weed
Becomes a flower: the lowliest reed
Beside the stream
Is clothed with beauty; gorse and grass
And heather, where his footsteps pass,
The brightest seem.

He sings of love, whose flame illumes
The darkness of lone cottage rooms;
He feels the force,
The treacherous undertow and stress,
Of wayward passions, and no less
The keen remorse.

At moments, wrestling with his fate,
His voice is harsh, but not with hate;
The brushwood hung
Above the tavern door lets fall
Its bitter leaf, its drops of gall,
Upon his tongue.

But still the burden of his song
Is love of right, disdain of wrong:
Its master chords
Are manhood, freedom, brotherhood;
Its discords but an interlude
Between the words.

And then to die so young, and leave
Unfinished what he might achieve?
Yet better sure
Is this than wandering up and down,
An old man in a country town,
Infirm and poor.

For now he haunts his native land
As an immortal youth; his hand
Guides every plow;
He sits beside each ingle-nook;
His voice is in each rushing brook,
Each rustling bough.

His presence haunts this room to-night,
A form of mingled mist and light,
From that far coast.
Welcome beneath this roof of mine!
Welcome! this vacant chair is thine,
Dear guest and ghost.

—*Henry W. Longfellow in Harper's Magazine.*

